# “Evaluation of physico-mechanical properties of naturally dyed betel-nut leaf plate (BLPF) – Banana blended fabric”

**DOI:** 10.1016/j.heliyon.2023.e13571

**Published:** 2023-02-10

**Authors:** Mohammad Naim Hassan, Moni Sankar Mondal, Naimul Hasan, Md Masum Reza, Md Ishtiaque Rahman, Joy Sarkar, Nourin Mohsin, Rafat Mahmud Hridoy, Dr Ahmed Jalal Uddin

**Affiliations:** a Department of Textile Engineering, Khulna University of Engineering & Technology, KUET, Khulna, 9203, Bangladesh; b Department of Yarn Engineering, Bangladesh University of Textiles, Dhaka, Bangladesh; c Department of Industrial Engineering and Management, Khulna University of Engineering & Technology, Bangladesh

**Keywords:** Banana-stem fiber, Betel nut leaf fiber, Waste material, Blended fabric, Natural dyes, Physico-mechanical properties

## Abstract

Betel-nut leaf plate fiber (BLPF) is a lingo-cellulosic natural fiber that can be used to make eco-friendly and biodegradable blended or hybrid fabric with Banana fiber. In the world of organic textiles, naturally dyed BLPF-Banana fiber can be used for wearable products and satisfy health and hygiene issues. BLPF and Banana fiber can be good natural fibers for hybrid fabrics despite being considered waste materials. In this research work, both of the fibers were pretreated carefully to get the desired fineness, color, flexibilities, etc., which are necessary to manufacture fabric. BLPF-Banana woven (1 × 1) hybrid fabric was developed where 12 Ne Banana yarns were used in the warp direction, and 20 Ne BLPF yarns were used in the weft direction and it was dyed naturally with Turmeric. Evaluations of different physico-mechanical properties; tensile strength (854.9 N), tearing strength (14.5 N), stiffness (3.1 N), crease recovery (75° angle), and fabric thickness (1.33 mm) of naturally dyed BLPF-Banana blended fabric were tested, and found satisfactory. SEM, FTIR, and Water vapor transmission tests were also conducted in this study. It attempted to turn the wastages into an asset to make a unique biodegradable BLPF-Banana hybrid fabric by blending two types of natural fibers with the help of natural dyeing substance; it could be a god replacement for synthetic blended fabric.

## 1 Introduction

Natural fiber can be collected from different sources like animals, vegetables, or mineral sources. These natural fibers can be used as textile fibers depending on their staple length and mechanical strength. Higher the staple length better the fiber quality [21]. Natural fibers are eco-friendly due to their biodegradable properties [1]. When two or more component fibers are associated with a single fabric, the specific fabric is called blended fabric. Generally, synthetic, and modified fibers are used to prepare the blended fabric. Synthetic fibers are produced by polymerization technique with the monitoring of different quality parameters [22]. The basic problem of using synthetic fiber is the non-degradable. However, the natural fibers are very likely to get attacked by microbial and can easily get degraded. Especially the cellulosic fibers can be attacked by aerobic bacteria and can cause strain on the fibers and physical deformation [2]. In order to prevent any unwanted degradation of the natural fibers, it is important to modify them by using modern chemical finishes [3]. However, it is also essential to process the natural fiber and remove the excess hemicellulose, lignin, pectins, and waxes from the fiber before using them [4]. To attain the proper functionality and higher strength-the natural fibers could be processed with suitable chemicals to enhance the overall performance.

In this research work, the main focus is on Banana fiber and Betel nut leaf plate fiber (BLPF). Both fibers used in this research are cellulose-based natural fibers and have a long staple length. Another common factor between these two fibers is that they have a high percentage of lignin in their raw form, making them much stiffer. So they are called lingo-cellulose fiber [5,6]. Both the Banana fiber and Betel nut fiber are abundant in nature and are often treated as waste material. This study is also a key factor in using natural waste materials and developing useful products. In case of Banana fiber, it is mainly collected from the pseudo stem of the Banana plant [7]. A process called Tuxing is used to collect the banana fiber from the Banana pseudo stem. The main elements of the Banana fibers are 61.50% α-cellulose, 20.30% hemicellulose, 15% lignin, and 3.2% other matters [8,9]. So, it can clearly be understood the lignin percentage in the Banana fiber is much higher, increasing its hardness. Moreover, the moisture regain percentage of Banana fiber is around 13%, indicating it can easily absorb the excess moisture from its surroundings [10].

On the other hand, the BLPF was collected from the Betel nut leaf plate. Betel nut leaf plate is mainly a hard shell-like material at the bottom of a leaf. When a leaf gets old enough, it falls off the tree by itself and can be collected for further processing [11]. Generally, it is considered as waste material and used firewood at the domestic level. The construction of the BLPF is 74–75% α-cellulose, 11–12% hemicelluloses, 9–10% lignin, and 2–3% other matters [12]. So the BLPF also needs to undergo the delignification process to remove the excessive portion of lignin from the raw fiber.

At the beginning of this research work, the Banana fiber was collected from the Banana pseudostem using the Tuxing method. Then it went through the delignification process using a high NaOH concentration. On the other hand, the BLPF was collected from the Betel nut leaf plate and went through the delignification process [13]. When both fibers were ready, Banana yarn and Betel nut yarn were prepared using a handloom. A 1 × 1 plane woven fabric was then made in a handloom using the Banana yarn (12 Ne) in the warp direction and the Betel nut yarn (20 Ne) in the weft direction. After preparing the raw Banana-betel nut blended fabric, it went through pretreatment processes. Scouring and bleaching were done to the raw fabric in the pretreatment processes.

Dying is a process by which coloring materials such as dyes, and pigments are applied to textile materials such as fiber, yarn, or fabric to achieve the desired color [23]. Traditionally fabric dying is mostly used in the textile industry. Synthetic dyes or artificial colors are widely used in the textile industry due to their low cost, availability, and outstanding coloring properties [24]. But synthetic dyes can be detrimental to the environment in various ways. As it contains azo group, sulfur, and other chemical groups which is harmful to the environment. As most of the coloring waste goes to the environment without any processing, it causes various kinds of environmental pollution such as acidification and water pollution, and overall hamper the development of aquatic life.

To minimize the pollution caused by synthetic dyes, industries are trying to find and use alternatives to synthetic dyes. And in that case, natural dyes can be a great alternative, as natural dyes come from natural sources and are easy to extract and process, and do not require any synthetic chemicals. As it comes from natural sources, natural dyes are environmentally friendly. Some of the natural dyes are onion dyes, dahlia, beet-root, lemon grass, turmeric, etc. As it does not possess any synthetic chemicals when it goes to the environment as waste after dyeing, it does not hamper or degrade the water quality like synthetic dyes by affecting the pH, BOD, and COD. When the organic waste comes to the water it acts as food or nutrient and leads to algal blood which in term causes eutrophication. Eutrophication creates a chain reaction in the ecosystem and leads to changes of pH, low oxygen, and acidification [25]. All these circumstances reduce the growth of aquatic animals and cause mutation in oceanic animals. And when these fishes served as food, it enters the terrestrial or land-dwelling animal food chain. In this way, it pollutes and affects the entire ecosystem. The environmental negative consequence of colorization could be avoided by utilizing natural dyes.

The scouring removes the raw fabric's unwanted leftover materials [14]. The bleaching process removed the natural color of the raw Banana-betel nut blended fabric to make it ready for natural dyeing [14]. In this research work, the main focus was to use the natural ingredients to make the Banana-betel nut blended fabric eco-friendly and cheap to produce on a mass scale. So, Turmeric was used as a natural dye in this research work [15]. Turmeric is a natural dye with has a bright yellowish ton. It is also an antibacterial agent which prevents the growth of any microbes on the surface of the fabric. It also has a good colorfastness property against dry and wet rubbing. Another important chemical that was used in this research work is Potassium Alum. This was used as a natural fixing agent to ameliorate the color quality of the dyed fabric [15]. After completing the natural dyeing process of Banana-Betel nut blended fabric, it underwent some physico-mechanical tests to evaluate the fabric. The yarn count of both Banana yarn and Betel nut yarn was measured to understand their fineness clearly. The thickness of the Banana-betel nut blended fabric was determined using the GSM test. GSM value of fabric indicates the thickness or bulkiness of that fabric [16]. EPI and PPI of the fabric were also determined to see how many Banana yarns were in a warp direction and how many Betel nut yarns were in an inch in the weft direction [17]. The thickness of the fabric was also determined. SEM test of the sample fabric was implemented to see the structure of the fabric in a very detailed view [18]. FTIR test was also carried out to identify the presence of different chemical bonds in the fabric [19]. Other tests like tensile strength, tear strength, crease recovery angle, and water vapor permeability were also measured better to understand the physico-mechanical properties of BLPF-Banana blended fabric. Banana-Betel nut blended fabric showed very promising physico-mechanical properties, which can be seen from those tests. The Banana-Betel nut blended fabric comes out as heavy fabric which can be used in various household purposes like table mats, curtains, carpet, sofa covers, pillow covers, bed sheets, etc. Various finishes can be used on this fabric for different technical purposes like medical, conductive, protective, etc.

Generally, CVC or PC fabrics are used as blended fabric. In CVC, the dominating percentage is cotton but in PC fabric-the maximum proportion is polyester. Apart from the traditional use, it would be a great choice to prepare blended fabric using the fully natural fibers in both parts of the fabric.

The objective of the study was to use natural fibers which normally useless and dumped into the environment- Betel nut leaf plate (BLPF), and banana fiber to prepare blended fabric together with the colorization as well as the evaluation of physico-mechanical properties of the prepared blended fabric, where the natural coloring source (Turmeric) was used. If the functionalities of the prepared fabric would be satisfactory, it could be a great replacement for synthetic blended fabric by fully natural blended fabric.

## 2 Materials and methodology

### 2.1 Materials

In this study, mainly Banana fiber and BLPF were used. Banana stem and Betel nut leaf plate were taken from the local areas of Khulna, Bangladesh. Various chemicals and natural materials were used in the fabric pretreatments, dyeing, and after-treatment processes. To carry out the pretreatment process, the chemicals, and auxiliaries; Detergent, sequestering agent, anti-creasing agent, anti-foaming agent, hydrogen peroxide, sodium hydroxide, stabilizer were utilized. The function of the used chemicals are stated- Detergent for cleansing, and reduction of surface tension of water, to remove the water hardness-sequestering agent was used, anti-creasing agent was used to prevent crease formation, to reduce foam generation-anti-foaming agent was utilized, to impart hydrophilicity, destruction of natural coloring materials-hydrogen peroxide, sodium hydroxide, stabilizer were utilized. The material (blended fabric) to be processed was fully prepared from a natural source; betel-nut leaf plate (BLPF), and banana fiber. In addition to that, the natural coloring material; Turmeric was used for colorization purposes. Distilled water was bought from Momotaj Chemical Ltd., Dhaka, Bangladesh. NaOH (>99%), H_2_O_2_(98.5%), Acetic Acid (CH_3_COOH) (M_w_ = 60.052 g mol^−1^) and Potassium Alum/KAl(SO_4_)_2_.12H_2_O were received from Sigma-Aldrich chemical Pvt. Ltd., India. Detergent, sequestering, anti-foaming, stabilizer, and leveling agents were also taken from Momotaj Chemical Ltd., Dhaka, Bangladesh.

### 2.2 Method

#### 2.2.1 Preparation of Banana fiber

The banana fiber was collected from the banana pseudostem. In order to collect the lingo-cellulosic banana fiber a decorticator machine was used. The leaves were stripped from the pseudostem and put into the decorticator machine. The fibers were separated from the pseudostem using a mechanical process known as the Tuxing process. After that, the raw banana fibers were cleaned thoroughly using sufficient water. Any residue or unwanted particles were washed off and the clean fibers were then dried under the sunlight. However, the banana fibers still had a high amount of lignin, which made them much stiffer. In addition, a large amount of water-insoluble gum and non-fibrous material was still present in the banana fiber, around 30–35%. So the delignification process was conducted. For this purpose, delignification process, an alkali solution was used. At first, the raw banana fiber was boiled in hot water for 1 h with NaOH (1 M solution). Hydrogen per oxide (H_2_O_2_) was used as a reducing agent ameliorate the delignification process. The fibers were extracted and then washed thoroughly with cold water. After that, the fibers were washed with 10% acetic acid solution for neutralization. When the neutralization process was over, a repeated washing of the fibers was carried out by cold water and dried in the drying machine for 1 h at 40 °C.

#### 2.2.2 Preparation of betel nut leaf fiber

Betel nut leaf fiber (BLPF) was collected from the Betel nut leaf plate. The leaf plate is normally a hard material due to excessive lignin. In order to make the leaf plate much softer, it was soaked into the water for 5 days at room temperature (25–30 °C). Afterward, the betel nut leaf fibers were separated manually from the plate by using the hand stripping method. Those raw fibers were then washed properly with sufficient water to remove the fibers' waste materials. Then the fibers were oven-dried at 40 °C for 24 h by using an oven drier. In order to prevent any further moisture absorption by BLPF, it was kept in the desiccators under a controlled atmosphere. However, the BLPF still had large amount of lignin in them. So an alkali treatment was done to remove the excess lignin from them. A 5 wt% solution of NaOH (Solid:liquid 5:100) was made using distilled water at room temperature from the alkali treatment process. The BLPF was then immersed in the 5 wt % of NaOH solution for 1 h. Afterward, the fibers were collected from the solution and washed thoroughly with sufficient water to remove any residual NaOH. After that, the fibers were washed with 10% acetic acid solution for neutralization. In order to remove the moisture from the alkali-treated BLPF, it was then oven-dried at 30 °C for 1 h.

#### 2.2.3 Preparation of Banana- betel nut leaf fibers blended fabric

**Fig. 1:**
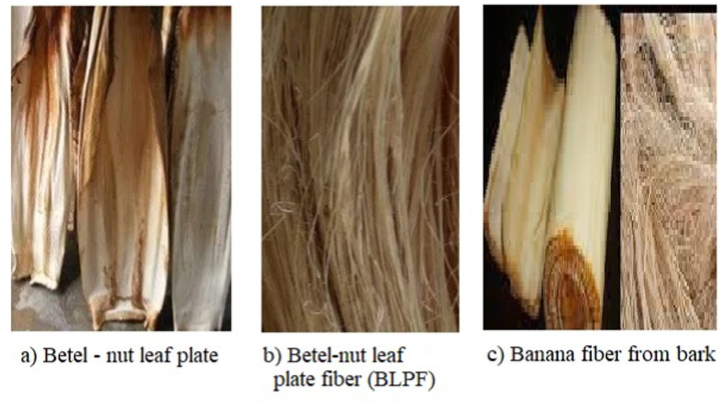
Utilized plant parts and fibers.

From the extracted Banana and Betel nut fibers, warp and weft yarn was developed, respectively. The hand spinning process was used to make the warp yarn from the Banana fiber and weft yarn from the betel nut leaf plate fiber. A 1/1 Plain woven fabric sample was prepared in a Handloom by using 50:50 Banana-Betel nut leaf fibers. Banana yarn with 12 Ne count was used in warp and Betel nut yarn with 20 Ne count was used in the weft position. The ends/inch of the fabric was 17 and picks/inch of the fabric was 15. Thus the raw fabric was produced and after that it went for the pretreatment process. The weave plain of the fabric is 1up, 1 down (see Fig. 1).

#### 2.2.4 Pretreatment of the fabric

**Fig. 2:**
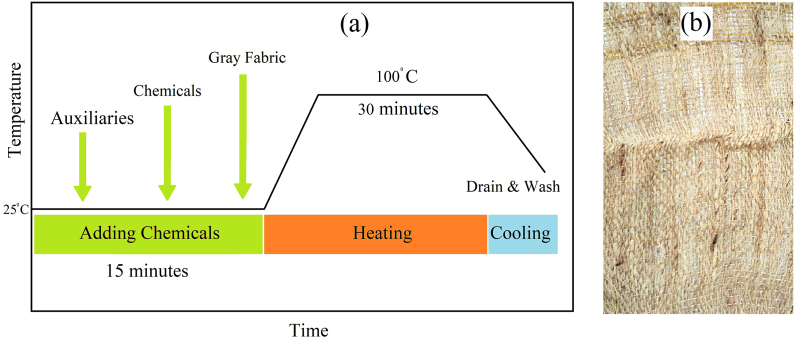
(A) Pretreatment process curve (b) Sample fabric after pretreatment process.

The prepared gray fabric was then gone through pretreatment where the scouring and bleaching were done to remove unwanted oil, wax and natural impurities from the fabric [26]. Various chemicals and auxiliaries were used in the pretreatment process. The liquor ratio of the pretreatment process was 1:40. According to the fabric weight, a sufficient quantity of water was taken into the machine's nozzle to make the solution. A detergent was used at the ratio of 1 g/L and added in to the solution. For removing the water hardness, sequestering agent was used at the rate of 0.5 g/L. An anti-foaming agent was also used at the rate of 0.2 g/L and added to the solution. NaOH was also used in the solution at the rate of 1.5 g/L. H_2_O_2_ was used as the bleaching agent and a stabilizer was used in order to prevent the break down on H_2_O_2_. The H_2_O_2_ and stabilizer were utilized at the rate of 2.5 g/L and 2 g/L, respectively. The chemicals and auxiliaries were added to the water and put inside the nozzle along with the sample gray fabric. After that, the nozzle was put in the sample dyeing machine. The pretreatment process was continued for 30 min at 100 °C. When the pretreatment process was done, the fabric was taken out and properly washed off with water. 10% acetic acid solution was used for neutralization, followed by a proper cold wash. The pretreated fabric is shown in Fig. 2(a), and the pretreatment process curve is shown in Fig. 2(b).

#### 2.2.5 Natural dyeing of pretreated fabric

**Fig. 3:**
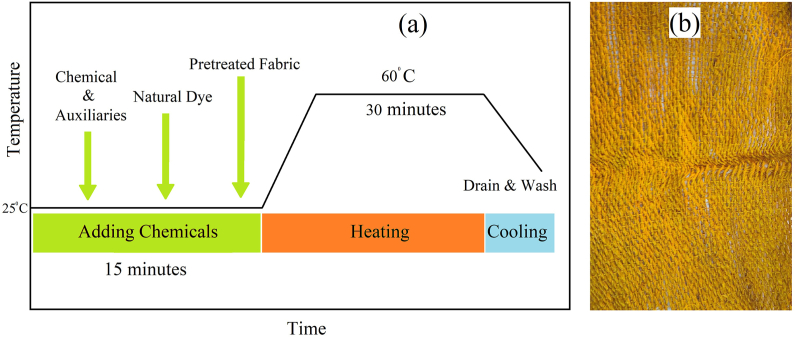
(a) Natural Dyeing process curve (b) Natural Dyed Fabric.

The pretreated fabric then went through a natural dyeing process with the help of natural dye (Turmeric), chemicals and auxiliaries. The liquor ratio of the dyeing process was 1:30. According to the fabric weight a sufficient quantity of water was fed into a nozzle to make the solution. For the removal of water hardness, sequestering agent was used at the rate of 0.5 g/L. In this process, the Turmeric liquid was used as the natural dye. In order to process the Turmeric, it was washed and cut into small pieces. Then it was blended with the presence of water. Then the solution was then filtered in order to remove any solid particles and collect the liquid Turmeric solution. The Turmeric solution was used at the rate of 15 g/L and a leveling agent was also added to the solution at the rate of 1 g/L to ensure the levelness of the dyeing process. Potassium Alum/KAl(SO_4_)_2_.12H_2_O was used at the rate of 5 g/L to increase the dye's durability. All the chemicals and auxiliaries were added in the water and put inside the nozzle along with the sample pretreated fabric. After that, the nozzle was put in the sample dyeing machine. The dyeing process was continued for 30 min at 60 °C. When the dyeing process was done, the fabric was taken out and properly washed off with water. 10% acetic acid solution was used for neutralization, followed by a proper cold wash. The naturally dyed fabric is showed is Fig. 3(a) and the dyeing process curve is shown in Fig. 3(b).

## 3 Result and discussion

The preparation, and testing sequence of the prepared blended fabric from natural resource are stated below as flow chart:

**Figure:**
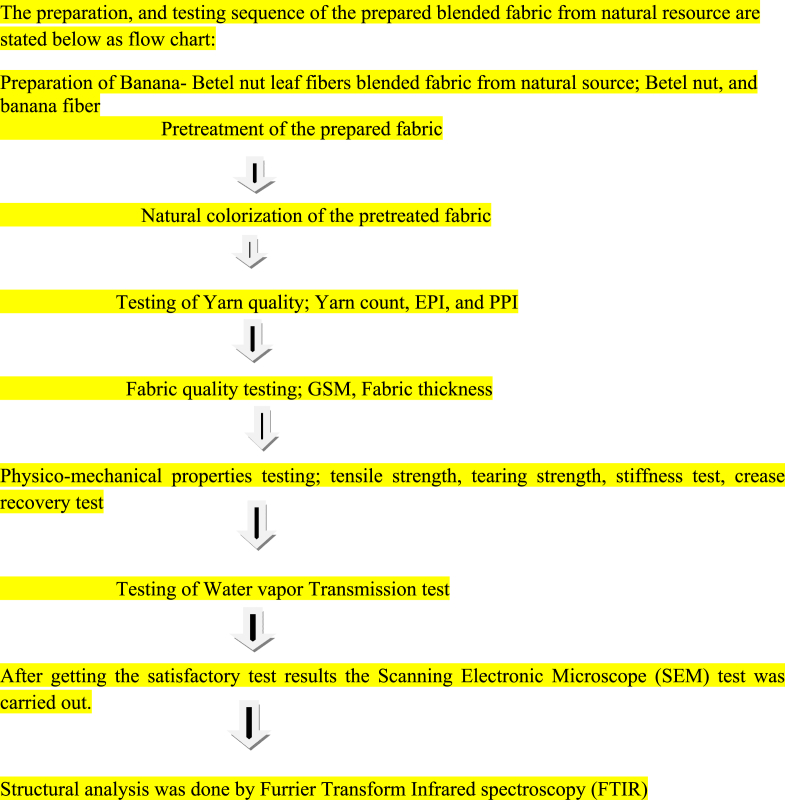

After completing pretreatment and natural dyeing of the prepared sample fabric, various tests were carried out to evaluate the physico-mechanical properties of the Banana- Betel nut leaf blended sample fabric. Descriptions of those tests and their results are analyzed below.

### 3.1 Yarn count

Yarn count indicates how coarser or finer the yarn is [27]. There are two methods to express the yarn count: direct method (weight per fixed length) and indirect method (length per fixed weight) [28]. The English count system (indirect system) was used in this research work. The formula for English count system is shown below.

N = (L × w) / (l × W)

Where, **N =** yarn count, **L =** the length of the sample, **w** = “unit of weight” of the system, **W** = the weight of the sample, **l =** “unit of length” of the system.

Yarn Count of the finished sample fabrics was determined three time. The test results are shown in Fig. 3. The warp yarn count and weft yarn count were at the desirable range. Warp count and weft count for sample 1 was 12Ne and 21 Ne respectively, warp count and weft count for sample 2 was 10Ne and 19 Ne respectively, and warp count and weft count for sample 3 was 9 Ne and 20 Ne respectively. So the average warp count was 12 Ne and average weft count was 20 Ne.

### 3.2 EPI and PPI

**Fig. 4:**
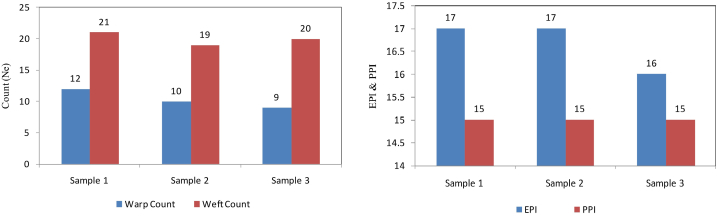
Warp count, weft count, and EPI, PPI test results.

EPI & PPI stand for “Ends Per Inch” and “Picks Per Inch” respectively [29]. Ends per Inch indicates how many Ends or warp yarn is there in 1 inch of a sample fabric in warp direction. On the other hand, Pick per Inch indicates how many Picks or weft yarn is there in 1 inch of a sample fabric in weft direction [30]. Three samples from three different fabric places were cut out to find the actual EPI and PPI of the sample fabric. The test results are shown in Fig. 4. The EPI and PPI were in the desirable range. Sample 1 had EPI and PPI of 17 and 15 respectively. Sample 2 had EPI and PPI of 17 and 15 respectively, and Sample 3 had EPI and PPI of 16 and 15 respectively. So the Average EPI and PPI was 17 & 15 respectively.

### 3.3 GSM

**Fig. 5:**
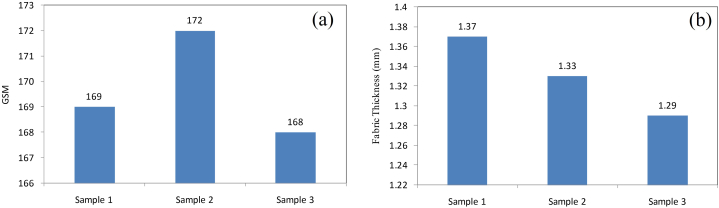
(a) Gsm test and (b) fabric thickness test results.

Fabric GSM test is mainly done to identify the thickness or thinness of the sample fabric [31]. ASTM D3776 test standard was used to measure the GSM value of the fabric. GSM of the finished fabric was determined three times as well. The GSM of sample 1 was 169, the GSM of sample 2 was 172, and the GSM of sample 3 was 168. So the average GSM value of the sample fabric was 170. This showed the fabric was a little bit coarser and suitable for heavy use. The graphical representation of GSM test results is shown in Fig. 5(a) below.

### 3.4 Fabric thickness test

Digital Thickness Gauge (meter) was used to determine the sample fabric's thickness. The ASTM- D1777 test standard was followed to conduct the test and repeated three times [32]. The test results were shown in the display of the meter using the mm unit. Fabric thickness test result data are shown below in Fig. 5(b). Sample 1 had a thickness of 1.37 mm, sample 2 had a thickness of 1.32 mm, and sample 3 had a thickness of 1.29 mm. So the average thickness of the fabric was 1.33 mm, which is suitable for various heavy purposes.

### 3.5 Tensile strength testing

Tensile strength test is necessary to know about the mechanical strength of the textile material [33]. A universal testing machine (UTM) was used for this test using the ASTM D638 standard. The sample size for conducting this test is 100 mm × 75 mm. A force of 500 N was applied in the warp direction of the fabric at a constant rate of expansion. This test set the constant rate of expansion to 75 mm/min. The testing condition was 25 °C temperature and 65% humidity. The Tensile tests for the finished sample fabric were done and the obtained results are shown below is Table 1. A tensile test was done for three times and the average test result was also determined.

**Table 1:** Tensile strength test results.

Test No	Force @ Peak (N)	Avrg.	Elong. @ Peak (mm)	Avrg.	Elong. @ Break (mm)	Avrg.	Strain @ Peak (%)	Avrg.	Strain @ Break (%)	Avrg.
1	856.8	854.9	23.81	24.14	31.44	30.58	11.90	12.07	14.78	15.29
2	848.3		25.77		28.61		10.81		17.93	
3	859.6		22.84		31.69		13.50		13.16	

**Fig. 6:**
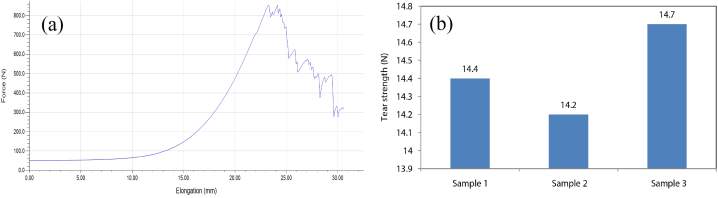
(a) Tensile Strength and (b) Tear strength test result curve.

From the UTM machine, a Force- Elongation curve was generated. The curve is provided below in Fig. 6(a).

From the findings, it can be observed that the finished fabric showed good Tensile strength-value 854.9 N force. The elongation at the peak was 24.14 mm in average. The elongation at break was 30.58 mm in average, which also indicated the good elongation property of the fabric. Strain at peak and strain at break were 12.07% and 15.29%, respectively.

### 3.6 Tearing strength test

Tear strength indicates the feasibility of a sample fabric resistant to tear along the deterioration position while facing external mechanical forces [34]. In this research, we used 2 pounds of dead weight, and the ASTM D412 test standard was maintained. According to the test standard, the sample fabric size was 210 mm × 50 mm. Tear strength tests were also done three times for the sample finished fabric. Sample 1 had tear strength of 14.4 N, sample 2 had tear strength of 14.2 N, and sample 3 had tear strength of 14.7 N. The test results were shown in Fig. 6(b), and 14.5 N was the average tear strength of the sample fabric which was a good tearing strength for the sample fabric in comparison to other fabrics.

### 3.7 Pneumatic stiffness test

**Fig. 7:**
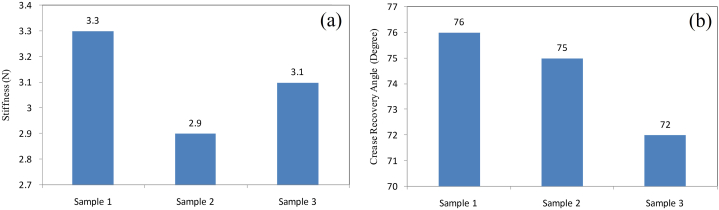
(a) Pneumatic stiffness test and (b) fabric crease recovery test results.

The pneumatic stiffness tester was used to evaluate a fabric's stiffness [35]. The pneumatic stiffens tester machine had a circular opening in the platform. The test sample size was 204 mm × 102 mm and placed on the platform. ASTM D4032 standard was used to measure the stiffness of the sample. The stiffness of the fabric was determined three times and the average result was determined, which was 3.1 N. Because sample 1 had a stiffness value of 3.3 N, sample 2 had a stiffness value of 2.9 N, and sample 3 had a stiffness value of 3.3 N. Stiffness test result data are shown below in Fig. 7(a). Test results show that the fabric has a moderate stiffness in compassion to other fabrics.

### 3.8 Crease recovery test

Crease Recovery properties indicate the ability to resistance against the formation of creases on the fabric's surface during mechanical pressure [36]. In the research work, the crease recovery angle of the finished fabric was determined using a crease recovery tester and the sample size was 15 mm × 40 mm. The crease recovery angle of the fabric was determined three times and the average result was determined. Crease recovery test result data are shown below in Fig. 7(b). Sample 1 had a crease recovery angle of 76°, sample 2 had a crease recovery angle of 75°, and sample 3 had a crease recovery angle of 72°. So the average crease recovery angle of the sample fabrics is 75°, indicating that the fabric has a better crease recovery ability.

### 3.9 Water vapor transmission test

The water vapor transmission test indicates the amount of water transmitted through the fabric surface area in a fixed amount of time [37]. In order to conduct the test, a Water-vapor Transmission Tester machine was used and the ASTM- F1249 test standard was followed. The humidity inside of the machine was 52% and the temperature was 32 °C. Then the machine ran for 10 min at that condition. After that, the container was taken out. The difference in the weight of the container before and after the test was determined.

Container and the Sample weight (before) = 176.977 gm, and (After) = 176.785 gm

Distilled water: 50 ml, Temperature: 32 °C, Humidity (%): 52%, Area: 0.4 ft^2^

Time: 10min = 0.17 h

Weight change= (176.977–176.785) = 0.192 gm

Water vapor transmission = {weight change ÷ (Time × Area)}

= {0.192 ÷(0.17 × 0.4)} = 2.824 gm/hr ft^2^

The test result shows that the water vapor transmission of the finished sample fabric was 2.824 gm/hr ft^2^ which is good for a fabric.

### 3.10 Surface morphology by scanning electronic microscope (SEM)

**Fig. 8:**
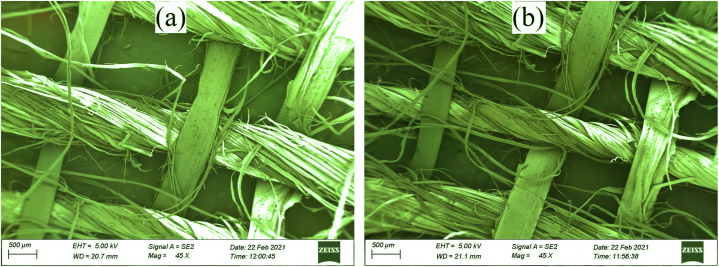
SEM image of sample fabric (a) Raw fabric (b) After Dyeing.

The scanning electron microscope (SEM) is used to observe the morphological pattern of a sample [38]. In this process, primary electrons are emitted on the sample and the final image is created by the secondary electrons detected in the machine. ASTM- F1372 test standard was utilized to carry out the SEM test using an SEM machine. The morphological structure of the BLPF-Banana blended fabric was checked using a scanning electron microscope (SEM) to observe the structural alterations in these objects. When the raw sample was studied at a specified magnification (45X), it was noticed that the neighboring raw fabric fibers were less compact, which suggested that the amorphous region was present in a large amount. When the m structural view of the dyed sample was checked, keeping the similar magnification fixed and compared to the view of the raw fabric, this was observed that the compactness in between the dyed sample fibers was increased. The colored sample had a higher level of crystallinity than the untreated sample. The strength of the cloth was positively impacted by the amorphous portion of the dyed sample being reduced. The SEM test results for raw and dyed fabric are shown in Fig. 8(a) and (B), respectively, below.

### 3.11 Structural analysis by Furrier Transform Infrared spectroscopy (FTIR)

**Fig. 9:**
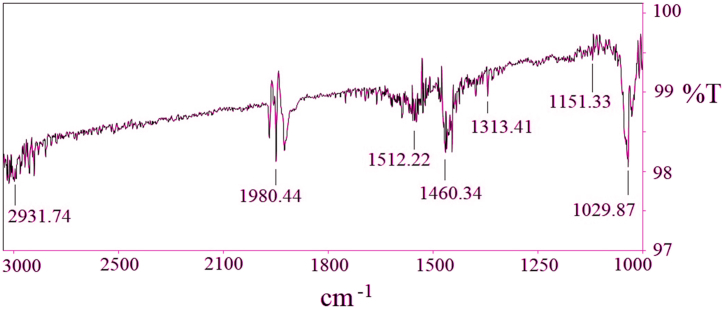
FTIR analysis of sample fabric.

In the FTIR test, the infrared region of the electromagnetic wave spectrum is analyzed in order to determine the presence of different bonds and chemical groups [39]. A small portion of the sample fabric was put inside an FTIR machine, and ASTM- E168 test standard was followed during the test. The sample fabric was exposed to infrared radiation, and the FTIR machine generated the absorbance graph. From the peaks at different positions in the curve, it can be determined which functional group and bonds are present in the sample fabric. The Test condition was strictly controlled at 25 °C and 65% Humidity. FTIR test was done to ascertain the physio-chemical characteristics of the dyed fabric sample. The FTIR absorbency graph for the dyed fabric is shown in Fig. 9.

The peak in the FTIR frequency graph at 1026 cm^−1^ suggests the stretching of C–O in the alcohol hydroxyl group [20]. The peak at 1029.87 cm^−1^ could be supported by the presence of similar stretching of C–O in alcohol hydroxyl group. The C–H bend stretching could be understood by the frequency range 1470-1450 cm^−1^. A similar C–H bend stretching was surmised from the peak at 1460.34 cm^−1^. The C=C stretching can be monitored by standard frequency range at 1680-1640 cm^−1^. A similar stretching was obtained from the peak at 1656.45 cm^−1^. The functional group; C–O is observed by the peak at 1382-1036 cm^−1^. A similar C–O functional group was monitored from the peak at 1313.41 cm^−1^ of tested sample. The existence of C–H functional group is shown from the frequency tolerance limit 2918.2–2954 cm^−1^. C–H functional group in the tested sample was ascertained from the frequency at 2931.74 cm^−1^ and 1980.44 cm^−1^. The existence of lignin was identified from the typical frequency range at 1514 cm^−1^. The presence of lignin was identified by the peak of the tested sample at 1512.82 cm-1 which was subsequently reduced after chemical treatment. The presence of cellulose, hemicelluloses, and oil is indicated by the standard frequency at 1175 cm-1 in the sample. The frequency at 1151.33 cm^−1^ could indicate the presence of such similarities in the tested fabric sample where the presented substances were reduced after several chemical treatments. It was elucidated from the FTIR test investigation that the tested sample was Banana- Betel nut blended fabric as both of them are natural fibers.

The Banana- Betel nut leaf blended fabric can be used in many sectors of textile products like diaper, napkin etc. As BLPF is naturally hygroscopic, it can be a good source of wearable cloths. On the other hand, it can also be used as floor mats, mattress cover, shopping bags etc. Further research should be conducted to improve the fineness of the warp and weft yarn to reduce the thickness and GSM of the fabric. This can help improve its hand feel and enable the fabrics to be used in various other fields.

In this study, the naturally dyed natural blended fabric was tested for evaluating the color fastness quality. The rating was 2–3 on the gray scale which indicated good fastness quality, but the color quality could be degraded in hot water washing (temperature greater than 100° Celsius). This deterioration could negatively influence the physico-mechanical properties of the prepared fabric. So, further research could be helpful to sort out the issue.

Lab scale machines were utilized to prepare the natural blended fabric. The yarn could be finer as well as higher count, if the fibers collection, and fabric manufacturing technique was done by semi-automatic or fully automatic machine. The automatic processing technique could enhance the tensile, and tearing strength of the fabric. The compactness of the fabric could be increased by incorporating more twists which could enhance the GSM of the fabric. These additional suggestions could improve the further functionalities of the blended fabric prepared from fully natural resources; Betel nut leaf plate (BLPF), and banana.

Different research was carried out on banana, and betel-nut leaf plate (BLPF) fiber [40]. In most of the cases, the study highlighted individual fibers or the optimum outcome was to prepare composites by using these two specific fibers. Apart from these, in this study, the blended fabric was prepared by using the fully natural fiber [banana, and betel-nut leaf plate (BLPF)] with satisfactory color, Yarn quality, and physico-mechanical properties; the average tensile strength was 854.9 N, tearing strength was 14.5 N on an average, stiffness average value was 3.1 N. The average fabric thickness was 1.33 mm. The crease recovery angle was 75° which indicated a better crease recovery ability, and the water vapor transmission test was up to a mark to be considered.

The compactness of the dyed fabric was higher than the untreated sample, which indicated the better crystallinity observed by the scanning electron microscope (SEM).

## 4 Conclusion

Ligno-cellulosic BLPF fibers were normally coarser and satisfactory fineness was achieved through pretreatments with NaOH. Yarns were made from both of these fibers to manufacture a woven cloth with a sample weaving machine. Both types of spinning; Ring and Rotor can be used to produce yarns from BLPF and Banana fibers. BLPF can be a good source of natural fibers as betel nut trees can be found in many regions that can easily be blended with other natural fibers. In this study, the main goal was to study the blended fabric and analyze this hybrid fabric's physico-mechanical properties. BLPF- Banana blended fabric showed satisfactory results as well. This fabric demonstrated necessary properties for use in wearable fabrics, agro textiles etc. The GSM, tensile strength, tear strength, stiffness and crease recovery test results were acceptable. As both of these fibers are hydrophilic products made from it, they will also be comfortable and dyed easily with natural and synthetic dyes. Water vapor transmission and SEM test results were good enough to find good dimensional stability of the fabric. The overall functional properties indicated the satisfactory outcome of the prepared natural blended fabric. The attempt to attain this kind of fabric could be a great impact on fabric processing considering the fully natural ingredients point of view.

## Author contribution statement

Mohammad Naim Hassan: Conceived and designed the experiments; Performed the experiments; Contributed reagents, materials, analysis tools or data; Wrote the paper.

Moni Sankar Mondal: Performed the experiments; Analyzed and interpreted the data; Contributed reagents, materials, analysis tools or data.

Naimul Hasan, Md. Ishtiaque Rahman, Ahmed Jalal Uddin: Analyzed and interpreted the data; Wrote the paper.

Md. Masum Reza, Rafat Mahmud Hridoy: Performed the experiments, Contributed reagents, materials, analysis tools or data.

Joy Sarkar, Nourin Mohsin: Contributed reagents, materials, analysis tools or data; Wrote the paper.

## Funding statement

This research did not receive any specific grant from funding agencies in the public, commercial, or not-for-profit sectors.

## Data availability statement

No data was used for the research described in the article.

## Declaration of interest’s statement

The authors declare no competing interests.
